# Montmorillonite—Eco-friendly and Effective Catalyst in the Synthesis of Biologically Active Compounds with Bicyclo[3.3.1] Moiety

**DOI:** 10.1021/acsomega.5c10876

**Published:** 2025-12-13

**Authors:** Eva Vrbková, Lucie Stoupová, Eliška Vyskočilová

**Affiliations:** Department of Organic Technology, 52735 University of Chemistry and Technology Prague, Technická 6, 166 28 Prague, Czech Republic

## Abstract

Prins cyclization is a key method for synthesizing oxygen-containing heterocycles with biological activity involving the reaction of alkenes and aldehydes under mild acidic conditions. This process is valuable for producing compounds such as 2,2,6-trimethyl-4-(1-propenyl)-3-oxabicyclo[3.3.1]­non-6-ene, a bioactive bicyclic ether synthesized from limonene and crotonaldehyde. Compounds with bicyclo[3.3.1]­nonene structures are interesting due to their fragrance properties and potential estrogen receptor activity. This study evaluates montmorillonite (MMT), a low-cost, environmentally friendly clay, as a heterogeneous acid catalyst for this reaction. Acid treatment of MMT (treated with HNO_3_, HCl, H_2_SO_4_, and H_3_PO_4_) had a positive influence on limonene conversion compared with nonmodified MMT, and limonene conversions >85% and selectivity >70% were obtained (24 h). Reaction parameters such as temperature, solvent, catalyst amount, and limonene-to-crotonaldehyde ratio significantly influenced conversion and selectivity. Higher temperatures and lower crotonaldehyde ratios improved the achieved limonene conversion, though selectivity decreased with temperature. Montmorillonite-supported heteropoly acids (HPW and HPMo) increased conversion but reduced selectivity and showed inefficient catalyst utilization at higher loadings. The initial reaction rate increased and selectivity decreased with catalyst acidity. Overall, acid-treated MMTs are more effective and economical than heteropoly acid-modified variants, offering a viable path for synthesizing bicyclic ethers via green chemistry.

## Graphical abstract

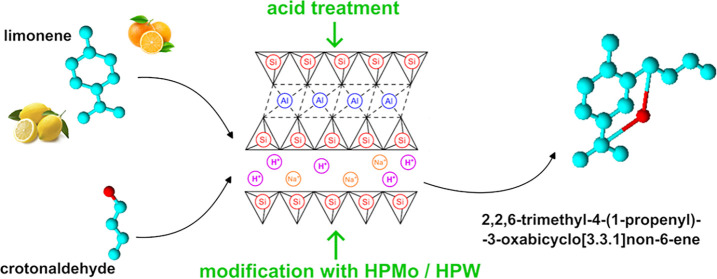

## 1 Introduction

Prins cyclization (cycloaddition) is a well-known synthetic process for the formation of different oxygen-containing heterocyclic molecules with biologically active properties. Prins cycloaddition (the reaction of a compound containing a double bond and an aldehyde) allows the creation of different heterocyclic compounds by creating new C–C or C-heteroatom bonds using quite mild synthetic conditions. Cyclization reaction is performed via oxocarbenic ion (from aldehyde), which attacks the π-bond (of the second reactant), and during this process, a new C–C bond is created. A Brønsted or Lewis acid can serve as a catalyst for this reaction. In recent years, an increased impact of the transformation of natural terpenes into biologically active molecules was observed.^1–3^ 2,2,6-Trimethyl-4-(1-propenyl)-3-oxabicyclo[3.3.1]­non-6-ene (TPOBN) is a potentially biologically active compound with great interest, which can be obtained from renewable sources, limonene or α-/β- pinene reacting with crotonaldehyde (Figure 1). The use of limonene in the Prins reaction provides a more straightforward reaction profile than α-pinene, often resulting in improved selectivity and a reduced formation of side products. Limonene is widely available not only from renewable citrus waste but also from alternative sources such as turpentine and tire pyrolysis oil. Its broader availability and biobased origin support its use in sustainable chemical synthesis. These factors make limonene a compelling substrate for catalytic transformations involving terpenes. Crotonaldehyde was selected as the second reactant due to its α,β-unsaturated aldehyde structure, which is highly reactive in acid-catalyzed electrophilic addition reactions such as the Prins reaction. Its conjugated system facilitates the formation of carbon–carbon bonds with terpenes, enabling the synthesis of functionalized products with potential applications in fine chemicals and materials. Moreover, crotonaldehyde is a relatively simple and commercially available aldehyde, making it a practical choice for evaluating catalyst performance and reaction selectivity under controlled conditions.

**Figure 1:**
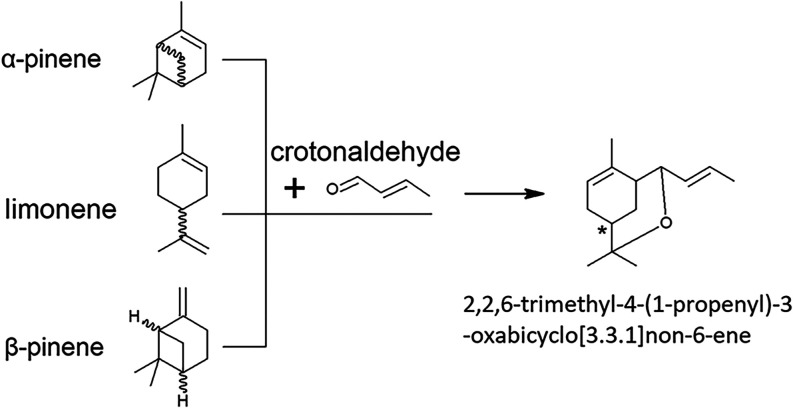
Reaction scheme of the Prins reaction of limonene or α-/β- pinene with crotonaldehyde.

Compounds with a bicyclo[3.3.1]­nonene moiety often have a desired sweet amberwood scent and are therefore used in the perfume industry to synthesize fragrances. Bicyclic ethers with bicyclo[3.3.1]­nonene moiety are described to have biological activity–interaction with estrogenic receptors α and β,^4,5^ TDP1 enzyme inhibition,^6^ antileishmanial properties,^7^ and potentially amantadine-resistant influenza A treatment.^8^ In the study of Costa et al.,^4^ synthesis of TPOBN was described using heterogeneous catalysts phosphotungstic acid (HPW) immobilized on silica or cesium salt of phosphotungstic acid in the reaction of crotonaldehyde with different terpenes (limonene, α-/β- pinene) with high terpene conversion (>95%) and high selectivity (94%). Salakhutdinov et al.^9^ reported the synthesis of TPOBN from limonene and β-pinene using askanite-bentonite clay in a yield up to 56%. Homogeneously catalyzed synthesis of TPOBN using iron­(III) chloride is also reported.^10^

Montmorillonite, a clay-based smectite material with a layered structure, is a well-known, cheap, environmentally friendly, and sustainable heterogeneous acid catalyst. The structure of montmorillonite consists of three layers, where the octahedral Al_2_O_3_ layer is located between two tetrahedral SiO_2_ layers. In the tetrahedral layer of SiO_2_, some Si atoms are replaced by Al^3+^, and in the octahedral layer of Al_2_O_3_, some Al atoms are replaced by Mg^2+^, causing the formation of anionic character. In the interlayer space, exchangeable cations (Na^+^, K^+^, Li^+^, Ca^2+^) are present.^13–17^ Application of montmorillonite as an effective acid catalyst is widely described, e.g., (i) in the Prins reaction—reaction of α-methylstyrene with paraldehyde, isoprenol with benzaldehyde or isovaleraldehyde, intramolecular cyclization of citronellal, and reaction of isopulegol with vanillin,^8–12^ (ii) in isomerization reactions—carvone to carvacrol, 1,2-limonenoxide to carvenone, and α-pinene to camphene,^18–20^ (iii) in water addition reactions—production of α-terpineol from α-pinene or hydration of styrene derivatives,^21,22^ (iv) in cyclization reactions—production of γ-valerolactone and synthesis of 4-chromanone,^23,24^ and (v) in esterification—production of fatty acid methyl esters or carvylacetate.^25–28^

This work aims to evaluate the catalytic activity of inexpensive and readily available montmorillonite in the Prins reaction of limonene with crotonaldehyde, a transformation that, to the best of our knowledge, has not yet been reported in the literature. Montmorillonite is well-known for its Brønsted acidity, making it an attractive candidate for acid-catalyzed transformations. Although the synthesis of TPOBN has previously been described using phosphotungstic acid and its salts,^4^ as well as askanite-bentonite clay,^9^ which is a natural form of montmorillonite, the systematic application of acid-treated montmorillonite in this specific reaction system has not yet been explored. Our study focuses on evaluating the effect of different acid treatments on montmorillonite and their influence on catalytic performance in the Prins cyclization of limonene. This study introduces a novel approach by employing montmorillonite in a previously unexplored reaction pathway and systematically evaluating its catalytic performance both as a standalone acid catalyst and as a support for heteropoly acids. Unlike prior work, which has focused primarily on conventional acid catalysts or complex supports, our research demonstrates that montmorillonite-based systems combine structural simplicity, low cost, and tunable acidity, offering a sustainable alternative to more expensive catalytic materials. The novelty lies not only in the direct application of montmorillonite but also in its dual role as an efficient support for heteropoly acids, providing new insights into catalyst design for the selective synthesis of 2,2,6-trimethyl-4-(1-propenyl)-3-oxabicyclo[3.3.1]­non-6-ene.

## 2 Results and Discussion

A large number of new materials were prepared by treatment with mineral acids (H_2_SO_4_, HNO_3_, HCl, and H_3_PO_4_) or by wet impregnation with different polyacids (phosphotungstic or phosphomolybdic acid) from commercially available montmorillonite K10. Activity of those materials was then evaluated in the Prins reaction of limonene with crotonaldehyde.

### 2.1 Material Characterization

Material composition was measured by X-ray fluorescence (XRF, Table 1). XRF results revealed in acid-treated montmorillonites a slight decrease in Al_2_O_3_ content (increase in Si/Al ratio), especially in the case of H_2_SO_4_/MMT, which could be connected with partial edges of Al_2_O_3_ octahedra leaching (described as a common result of acid treatment^29^). The content of K_2_O was stable, meaning probably its location in the structure, not in the interlayer space. A light decrease of MgO content was again probably connected with partial edges of octahedra leaching, as some of the Al^3+^ atoms in octahedra are often substituted with Mg^2+^ atoms.^30^ In the case of H_3_PO_4_/MMT, a small content (0.2 wt %) of P_2_O_5_ was observed, probably the residue from acid treatment. No correlation between acid p*K*_a_ and the decrease in Si/Al ratio was observed. In the case of phosphotungstic (HPW) and phosphomolybdic (HPMo) modified montmorillonites, the presence of WO_3_ respectively MoO_3_, and P_2_O_5_ was observed, proving successful modification by appropriate heteropoly acid (HPA).

**Table 1:** Material Composition—Results from X-ray Fluorescence

	XRF (wt %)									
material	Al_2_O_3_	SiO_2_	Fe_2_O_3_	MgO	K_2_O	MoO_3_	WO_3_	P_2_O_5_	others	Si/Al (mol)
MMT K10	12.1	80.9	2.5	1.5	1.6				1.4	5.7
H_2_SO_4_/MMT	10.1	84.1	1.5	1.1	1.6				1.6	7.1
HNO_3_/MMT	11.9	81.3	2.3	1.4	1.7				1.4	5.8
HCl/MMT	11.5	82.0	2.1	1.3	1.6				1.5	6.1
H_3_PO_4_/MMT	11.7	81.4	2.3	1.3	1.7			0.2	1.6	5.9
1HPMo/MMT	11.9	79.9	2.5	1.5	1.7	1.0		0.1	1.4	5.7
5HPMo/MMT	11.5	76.5	2.5	1.4	1.6	4.7		0.3	1.4	5.6
10HPMo/MMT	10.7	70.8	2.4	1.3	1.6	11.4		0.5	1.3	5.6
20HPMo/MMT	10.2	66.2	2.1	1.2	1.6	16.6		1.0	1.1	5.5
1HPW/MMT	11.9	79.8	2.5	1.5	1.6		1.2	0.1	1.5	5.7
5HPW/MMT	11.3	76.2	2.5	1.4	1.7		5.4	0.2	1.6	5.7
10HPW/MMT	10.6	70.9	2.3	1.3	1.5		11.7	0.4	1.7	5.7
20HPW/MMT	9.7	63.0	2.2	1.1	1.5		20.9	0.5	1.7	5.5

Nitrogen physisorption was used to compare the textural properties of materials (Table 2 and Figures S1–S3). All materials showed adsorption isotherms of type IV (IUPAC classification). Samples consisted mostly of only mesopores with classical type hysteresis loop H3 (IUPAC classification), which corresponded to nonrigid aggregates of plate-like particles (e.g., clays).^31^ In the case of acid-treated materials, the decrease of specific surface area was observed in the case of H_2_SO_4_/MMT (185 vs 254 m^2^/g for nonmodified MMT) and also in the case of HCl/MMT and H_3_PO_4_/MMT (214 or 234 m^2^/g, respectively). The decrease was in accordance with the increase of the Si/Al ratio determined by XRF except for hydrochloric acid (Figure S4). On the other hand, modification with HNO_3_ led to a slight increase of *S*_BET_ (262 m^2^/g). All acid-treated materials were mesoporous—the content of micropores was very low (max. 0.4%), and total pore volume was similar (0.32–0.39 cm^3^/g) in all cases, with the exception of H_2_SO_4_/MMT, which possessed the smallest pore volume (0.28 cm^3^/g) of all acid-treated materials. In the case of modification of materials with HPAs, a significant decrease in specific surface area was observed with increasing HPA content—for materials with 20 wt % HPMo, it was 133 m^2^/g, and for material with 20 wt % HPW, it was 158 m^2^/g, caused by the pore blockage by HPA. In the case of modification with HPAs, an increase in micropore volume (up to 3.2% in the case of HPMo and 4.3% for HPW) was observed also with increasing HPA amount. The micropores were probably formed in the structure of the HPA on the surface itself. That is why those were observed only after higher loadings of HPA. A slight decrease of total pore volume was also observed with increasing HPA amount.

**Table 2:** Textural Characteristic Measured by Nitrogen Physisorption and Acidity of Material Determined Using Temperature-Programmed Desorption of Ammonia

	nitrogen physisorption			temperature-programmed desorption (μmol_**NH3**_/g)		
	*S*_BET_ (m^2^/g)	*V*_p_ (cm^3^/g)	*V*_micro_ (%)	total	weak + moderate	strong
MMT	254	0.39	0.3	337	337	0
H_2_SO_4_/MMT	185	0.28	0.4	587	355	232
HNO_3_/MMT	262	0.39	0.3	580	291	289
HCl/MMT	214	0.32	0.3	551	321	230
H_3_PO_4_/MMT	234	0.36	0	563	231	333
1HPMo/MMT	219	0.35	0	552		
5HPMo/MMT	193	0.31	0.4	710		
10HPMo/MMT	172	0.29	1.2	782		
20HPMo/MMT	133	0.24	3.2	964		
1HPW/MMT	228	0.36	0	398		
5HPW/MMT	212	0.32	1.0	580		
10HPW/MMT	188	0.30	1.8	732		
20HPW/MMT	158	0.26	4.3	898		

Temperature-programmed desorption of ammonia was used to evaluate the acidity of materials. In the case of acid-treated montmorillonites, an increase in material acidity was observed after modification (from 337 to 551–587 μmol_NH3_/g). Acid treatment also led to the origination of new, strong acid sites (Figure 2), and a slight decrease in the amount of weak + moderate acid sites was observed. Determination between weak + moderate acid sites and strong acid sites at acid-treated montmorillonites was based on temperature ranges of NH_3_ desorption in TPD profiles. Weak + moderate acid sites were attributed to 50–350 °C and strong acid sites to 350–650 °C. The amount of new strong acid sites increased in row, HCl/MMT ∼ H_2_SO4/MMT < HNO_3_/MMT < H_3_PO_4_/MMT, which was in accordance (except hydrochloric acid) with decreasing p*K*_a_ of acids used for treatment. The highest value of strong acid sites in H_3_PO_4_/MMT can be affected by the presence of residual phosphoric acid, detected by XRF analysis (Table 1). The temperature maximum for weak + moderate acid sites was around 140 °C and for strong acid sites was around 550 °C. The prevalent acid sites in MMT are the Brønsted acid sites.^32^ The Brønsted acid sites in acid-treated MMT are from (i) interlayer H_3_O^+^, (ii) protonation of SiO groups in the layers of MMT due to the breakage of Al–O–Si bonds after acid treatment, and (iii) a proton attraction by hydroxyl groups Si–OH and Al–OH in an acid environment.^20,33,34^ The unsaturated Al^3+^ ions present at broken edges of MMT might be the source of Lewis acidity.^20^ For HPA-modified montmorillonites, only a single broad desorption peak was observed in the temperature-programmed desorption (TPD) spectra (Figures S5 and S6). This peak did not allow a clear distinction between weak and strong acid sites; therefore, only the total acidity is reported for these samples. In the case of HPA modification, a significant increase of material acidity with increasing HPA amount was observed—material with 20 wt % HPMo had acidity of 964 μmol_NH3_/g and material with 20 wt % HPW had 898 μmol_NH3_/g, which was more than double the value of the original MMT K10 acidity.

**Figure 2:**
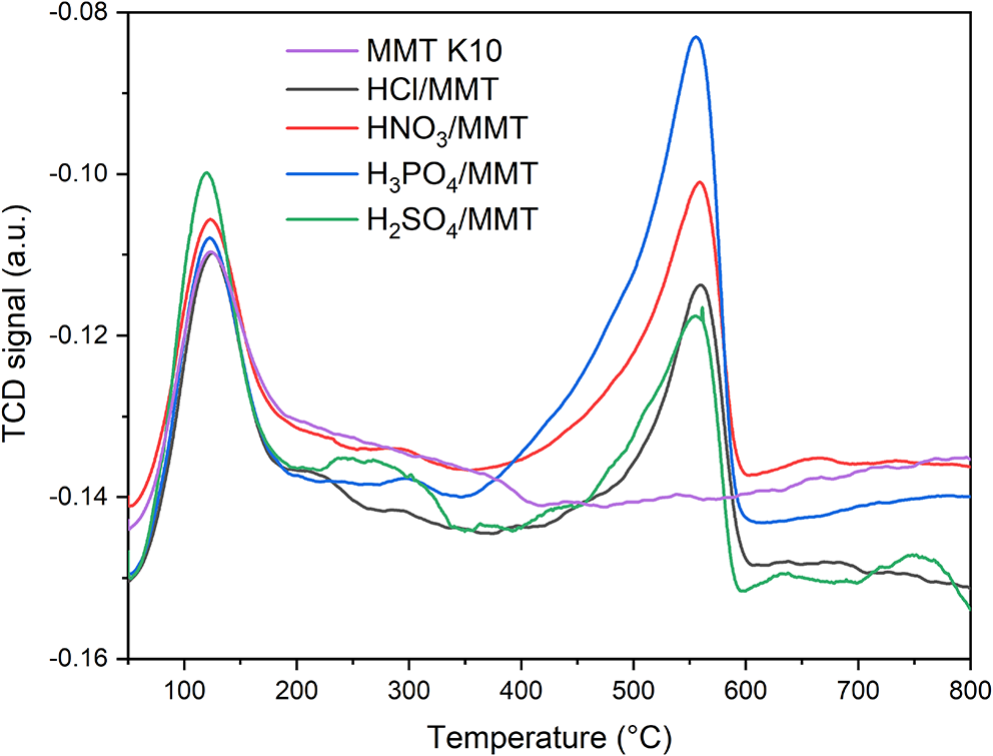
Temperature-programmed desorption of ammonia for acid-treated montmorillonites.

DR-UV–vis was used to compare prepared materials (Figure S7). Nonmodified MMT K10 possessed one absorption maximum around 250 nm.^23,35^ Maxima for HPW was 265 nm, and at low loadings, it was covered by the band of MMT. At high loading, the prevailing band corresponded mainly to HPW that covered the surface of MMT. Different observed behavior of both acids on MMT was given by the different absorption properties of HPMo compared with HPW. At high loading of HPMo, a significant decrease of the band at 253 nm was observed, and the band at 320 nm corresponding to HMPo was present. It can be concluded that the surface of MMT was fully covered by HPMo.

ATR-FTIR of acid-treated MMT showed^36^ (Figure S8) the presence of bands corresponding to deformation vibration of Si–O–Al (at 530 cm^–1^), vibration of Al–Fe–OH (at 850 cm^–1^), Si–O–Si asymmetric valence vibrations (1060 cm^–1^), and a strong band corresponding to bending vibrations of inner OH groups in octahedra (1300 cm^–1^). Also, the band typical for valence vibration of hydroxyl groups at 3600 cm^–1^ was present together with weak valence vibrations of adsorbed water (3400 cm^–1^). Overall, the spectra were typical for the MMT structure. Only the slight change in spectra after treatment by acids was the more visible band (shoulder of band at 1300 cm^–1^) at 1620 cm^–1^ corresponding to interlamellar water present in a higher amount after acid treatment.

MMT with loaded HPMo possessed (Figure S9) all of the bands typical for montmorillonite, accompanied by bands of HPMo (1200 cm^–1^ P–O vibration, connected with the structural integrity, 1060 cm^–1^ P–O vibration of central PO_4_ unit, strong band at 820 cm^–1^ Mo–O–Mo vibration and 730 cm^–1^ P–O–Mo vibration), confirming the successful loading of phosphomolybdic acid and the remnant of its Keggin structure.^37^ The intensity of the bands increased with increasing the amount of HPMo loaded. A slight shift of typical HPMo bands confirmed the interaction with montmorillonite.

Spectra of MMT loaded by HPW also had typical bands for montmorillonite and HPW^38^ that were slightly shifted as a result of the mutual interaction (Figure S10). The intensity of HPW bands increased with the increasing amount of loaded HPW. The corresponding bands were 1080 cm^–1^ (P–O vibration of central phosphorus) and 890 cm^–1^ (W–O–W vibration of corner oxygen). The bands corresponding to the W=O terminal and W–O–W edges were hidden behind the strong band at 850 cm^–1^ montmorillonite. However, based on literature,^39^ we can say that the Keggin structure of HPW remained intact, the same as MMT layers.

### 2.2 Catalytic Testing

The above characterized materials were used as catalysts for the preparation of 2,2,6-trimethyl-4-(1-propenyl)-3-oxabicyclo[3.3.1]­non-6-ene (TPOBN) by Prins reaction of limonene with crotonaldehyde. Montmorillonite was treated by mineral acids or used as a support for two heteropoly acids, and both types of materials were compared to evaluate their behavior in the reaction. The correlation between material properties and their efficiency as catalysts was evaluated, and the reaction conditions were optimized to obtain the highest yield of the desired compound.

#### 2.2.1 Acid-Treated Montmorillonites

The influence of the type of catalyst prepared by using different acids for treatment on the reaction course was performed (Figure 3 and Table 3). We observed that any acid treatment led to a significant increase of catalytic activity compared with nonmodified MMT K10. Considering the fact that commercially available MMT K10 is modified with hydrochloric acid itself,^40^ it was a little surprising. However, the absence of strong acid sites in MMT K10 was already observed not only in our previous works^9^ but also reported by others.^41^ So, the follow-up treatment of our original MMT K10 deserved our attention. The highest conversion (90% conversion, 70% selectivity, 24 h) was obtained using HNO_3_/MMT. Its behavior could be connected with its highest specific surface area (262 m/g) of all acid-treated materials because the acidity was comparable for all prepared materials. On the other side, HNO_3_/MMT contained the highest amount of Al^3+^ from all acid-treated materials (the lowest ratio Si/Al), which can also be responsible for its higher activity; however, the conversion course using all of them was almost the same. The highest leaching of Al from the structure in the case of H_2_SO_4_/MMT (the highest ratio Si/Al) influenced neither selectivity nor conversion. Selectivity did not change significantly with conversion and was similar for all materials (70–82%). The highest selectivity (82%, 24 h) was obtained using H_3_PO_4_/MMT. However, the difference in the yield was in the range of measurement error (63% HNO_3_/MMT, 70% H_3_PO_4_/MMT). Nevertheless, the prolonged time could lead to increased conversion and the same selectivity in the case of H_3_PO_4_/MMT, thus the yield would be higher. In the case of HNO_3_/MMT, we observed the highest initial reaction rate of 4.9 mmol/g·h, which was almost 3.3 times higher than the value of the initial reaction rate for nonmodified montmorillonite (1.5 mmol/g·h) (Table 3, rows 1–5).

**Table 3:** Conversion, Selectivity, and Initial Reaction Rate under Reaction Conditions Screening Using Acid-Treated Montmorillonites (Molar Ratio L:C = 1:1.5, 24 h)

row	material	solvent	temperature (°C)	catalyst amount (wt %)	conversion (%)	selectivity (%)	initial reaction rate (mmol/g·h)
1	MMT	toluene	60	80	72	77	1.5
2	HNO_3_/MMT				90	70	4.9
3	HCl/MMT				85	76	3.7
4	H_3_PO_4_/MMT				85	82	2.9
5	H_2_SO_4_/MMT				86	74	2.7
6			40	40	19	94	0.8
7			60		57	78	4.3
8			80		68	69	8.6
9		1,4-dioxane	60		67	58	5.3
10		heptane			14	63	1.0
11		acetonitrile			90	0	0.0

**Figure 3:**
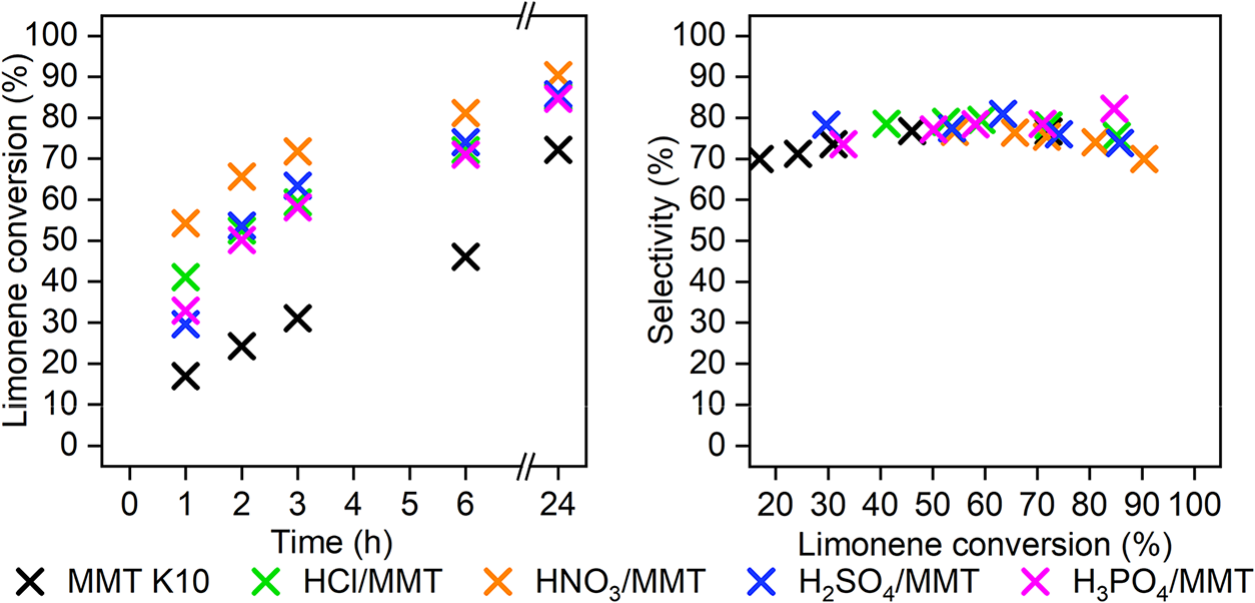
Reaction course in the case of using different acid-treated montmorillonites (toluene, 80 wt % cat. to limonene, molar ratio L:C = 1:1.5, 60 °C).

The influence of temperature on the reaction course was studied using H_2_SO_4_/MMT as a catalyst and toluene as a solvent (Figure 4). It can be observed that with increasing reaction temperature, the higher limonene conversion at 24 h and initial reaction rate were obtained (Table 3, rows 6–8). Using 40 °C, an increasing trend of selectivity on conversion was observed; on the other hand, using 60 and 80 °C, the selectivity did not change significantly with limonene conversion. At a reaction temperature of 40 °C, a noticeable increase of selectivity with respect to limonene conversion was observed. In contrast, at elevated temperatures of 60 and 80 °C, selectivity remained relatively constant across the range of conversions studied. The observed increase in selectivity at 40 °C appeared to be associated with the rapid formation of side products, most likely through aldol condensation of crotonaldehyde or isomerization of limonene. At higher conversions, protonated crotonaldehyde and protonated limonene were effectively utilized in the desired Prins reaction, which accounts for the observed improvement in selectivity. However, it was clear that with increasing temperature, lower selectivity was obtained. The decrease in selectivity with increasing temperature was primarily linked to the formation of byproducts with a *p*-menthane structure derived from the starting limonene. The protonated form of limonene, which serves as an intermediate for both the desired product and these byproducts, appears to be less stable at higher temperatures. At elevated temperatures, thermodynamic control becomes increasingly dominant, favoring isomerization processes over the kinetically preferred desired pathway. A significant increase of initial reaction rate with increasing temperature was observed—0.8 mmol/g·h for 40 °C up to 8.6 mmol/g·h for 80 °C. It was obvious from the reaction course (Figure 4) that conversion could increase with the prolongation of the reaction time. This possibility was tested, using 80 wt % HNO_3_/MMT, toluene, 60 °C, and L:C = 1:1.5 molar ratio, which led to 73% limonene conversion and 92% selectivity, 48 h (Figure S11). The apparent activation energy of this reaction was calculated based on the three temperature levels. Its value was 57 kJ/mol. The calculated value was higher than the value calculated for the Prins reaction of propylene with formaldehyde (15–34 kJ/mol),^40^ but lower compared with the Prins reaction of 3-carene with formaldehyde (76 kJ/mol),^15^ or α-pinene or β-pinene with formaldehyde (84 or 98 kJ/mol, respectively).^42,43^

**Figure 4:**
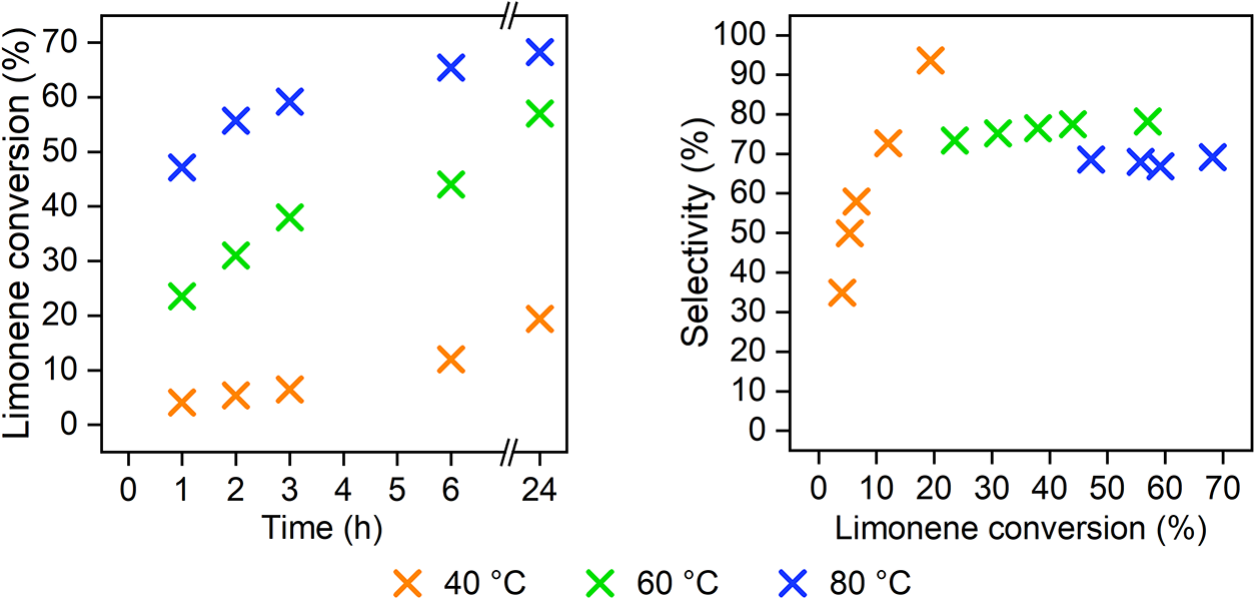
Reaction course in the case of using different reaction temperatures (toluene, 40 wt % cat. H_2_SO_4_/MMT to limonene, molar ratio L:C = 1:1.5).

Solvent influence on reaction course was tested; toluene, heptane, 1,4-dioxane (nonpolar aprotic), and acetonitrile (polar aprotic) were chosen for the reaction (Figure S12 and Table 3, rows 7, 9–11). Toluene and 1,4-dioxane provided similar limonene conversion (57 and 67%, 24 h), but different selectivity (78 vs 58%, 24 h). The initial reaction rate observed for toluene and 1,4-dioxane was 4–5 times higher compared with heptane. Using heptane, only a low limonene conversion (14%, 24 h) was obtained; selectivity was comparable to using toluene and 1,4-dioxane. The achieved conversion and initial reaction rate (with the exception of acetonitrile) seemed to be dependent on the solvent acidity described by p*K*_a_ (Figure 5).

**Figure 5:**
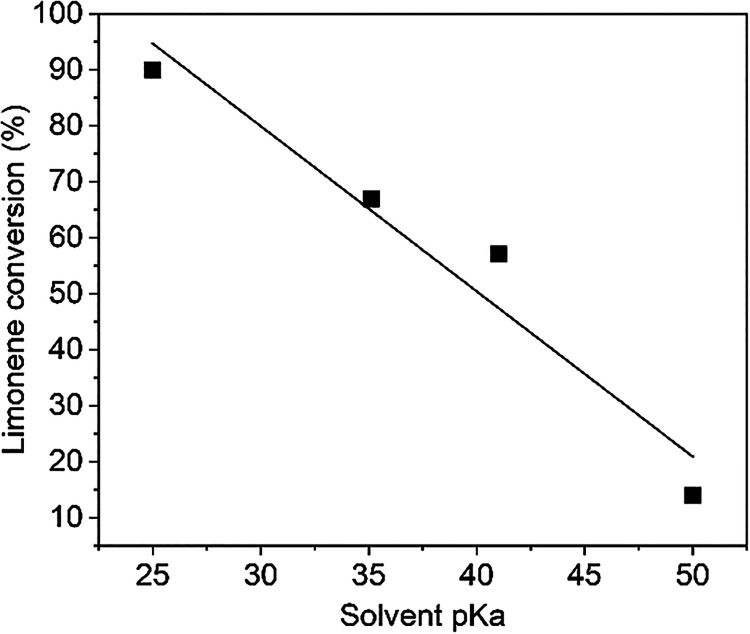
Dependence of limonene conversion and initial reaction rate on solvent p*K*_a_.

However, other solvent properties could also play a role. Using acetonitrile led to the highest limonene conversion among tested solvents (90%, 24 h), but selectivity to the desired bicyclic ether with bicyclo[3.3.1]­nonene moiety was 0%, because, surprisingly, only limonene oxide occurred in the reaction mixture. Moreover, the initial reaction rate (calculated in 1 h) was 0 mmol/g·h because a long initiation period was observed using acetonitrile. A different reaction course using acetonitrile can be explained by a different acetonitrile relative permittivity, which is 37.5 compared with 1.9–2.4 for other used solvents.^13^ Acetonitrile is also known to support the oxidations together with the properties of montmorillonite.^44,45^ Compared with aliphatic heptane using toluene and 1,4-dioxane, better results were obtained. In the case of an acid-catalyzed reaction, it can be connected with the stabilization of the carbenium cation intermediate by 1,4-dioxane by free electron pairs at the oxygen in the solvent structure.^13^ Interaction of the toluene aromatic ring (high p*K*_a_ value = 40.9) with reaction intermediates by π-interaction is also possible.^13^

The influence of catalyst amount was also tested in the range of 40–120 wt % using H_2_SO_4_/MMT and 60 °C (Figure S13). Using 80 and 120 wt % catalyst led to a comparable limonene conversion, which was higher than that for 40 wt % cat. amount (57% vs 86–92%), and selectivity remained the same for all catalyst amounts (70–80%). The study of reactant molar ratio was also performed (Figure S14); molar ratios L:C = 1:1.5, 1:2, and 1:3 were tested. Molar ratio L:C had a significant influence on limonene conversion and at selectivity. The highest limonene conversion was obtained using the lowest L:C ratio of 1:1.5, which can be explained by the dilution of the reaction mixture using a higher excess of crotonaldehyde. Selectivity obtained using all L:C ratios was comparable (78–83%, 24 h). The influence of reaction mixture dilution on the reaction kinetics was also confirmed by the initial reaction rate values; the highest value was observed for L:C = 1:1.5 (4.3 mmol/g·h). With increasing molar excess of crotonaldehyde, a decrease of the initial reaction rate was observed (3.8 and 2.2 mmol/g·h, respectively).

Among the acid-treated montmorillonites, the overall acidity of the catalyst did not significantly affect either the initial reaction rate or selectivity (Figure S15). For MMT, HCl/MMT, and HNO_3_/MMT, a linear trend was observed, where higher acidity correlated with increased reaction rates. However, this correlation was not evident for H_3_PO_4_/MMT and H_2_SO_4_/MMT.

When strong acid sites were considered separately, a similar lack of consistency was noted. Interestingly, for weak and medium acid sites (Figure S15), an opposite trend appeared: for HNO_3_/MMT, HCl/MMT, and H_2_SO_4_/MMT, increasing acidity was associated with a linear decrease in the reaction rate, while pure MMT and H_3_PO_4_/MMT deviated from this pattern. Regarding selectivity (Figure S16), no clear trend was observed with total acidity or strong acidity. Only in the case of weak and medium acid sites, a partial trend emerged; except for H_3_PO_4_/MMT and HNO_3_/MMT, selectivity decreased with increasing acidity.

These deviations may be attributed to several factors, including differences in acid site accessibility, pore structure alterations caused by acid treatment, and the presence of noncatalytically active surface species. In particular, phosphoric and sulfuric acid treatments may lead to structural changes or surface passivation, which could suppress expected catalytic behavior despite increased acidity.^46^

The possibility of catalyst reuse was tested (Figure S17), as it is the most important advantage of using a heterogeneous catalyst. A significant decrease of limonene conversion using recycled catalyst was observed (90% vs 35%, 24 h); on the other hand, the selectivity remained the same—70% at 24 h. The reason for the decrease of limonene conversion using recycled catalyst can be explained by the presence of carbonaceous deposits on the material surface after using in reaction. The presence of carbonaceous deposits was confirmed by temperature-programmed oxidation, and this analysis showed that recycled catalyst contained 10.6 mg_C_/g_mat_ (no carbonaceous deposits were present on the fresh catalyst). Temperature-programmed analysis revealed a broad peak with a local maximum in the range of 400–500 °C. Based on this observation, the regenerated catalyst was calcined at 500 °C and subsequently treated with acid to restore its structure.^18^ This regeneration process resulted in a partial recovery of the material’s catalytic activity (70% limonene conversion, 24 h) while maintaining a selectivity of approximately 70%. Nevertheless, only the partial loss of activity seemed to be promising for further utilization of our materials in the studied reaction. Additional research focused on the conditions of catalyst regeneration would be beneficial.

### 2.3 Heteropoly Acid-Modified Montmorillonites

Two types of heteropoly acids were used for MMT modification: phosphotungstic and phosphomolybdic acids. Both of them were successfully impregnated on clay support, characterized, and used as catalysts in the studied Prins reaction. The reaction without any catalysts did not show any activity.

Using HPW-modified montmorillonites revealed an increase of limonene conversion (78–91%, 24 h) and initial reaction rate (1.8–5.5 mmol/g·h) with increasing HPW amount on the catalyst (Figure S18 and Table 4, rows 2–5). Selectivity was similar, independent of HPW content (68–76%, 24 h). There was an increase of initial reaction rate from 1.8 mmol/g·h for material modified with 1 wt % HPW to 5.5 for material modified with 20 wt % HPW. However, the reaction rate related to the HPW amount on the montmorillonite support showed that HPW was not efficiently used for catalysis. Even more, the reaction rate was lower compared with pure MMT, meaning that its surface was covered by HPW and probably was not active in catalysis. Thus, 1HPW/MMT possesses a significantly higher specific activity compared with 20HPW/MMT. While 20HPW/MMT provided a specific initial reaction rate of 0.28 mmol/g·h, 1HPW/MMT provided a significantly higher value, 1.8 mmol/g·h. Even if 20HPW/MMT contained a higher amount of HPW in material, its real utilization was ca. 7 times lower compared with 1HPW/MMT, meaning that not all HPW species were accessible for the reaction.

**Table 4:** Reaction Course in the Case of Different Reaction Conditions Using HPA-Modified Montmorillonites (Molar Ratio L:C = 1:1.5, 80 wt % cat. to Limonene, 60 °C, 24 h, HPW and HPMo Calculated to Be Corresponding to the Amount of HPA in 10HPA/MMT)

row	material	conversion (%)	selectivity (%)	initial reaction rate (mmol/g·h)	specific initial reaction rate (mmol/g·h)
1	MMT	72	77	1.5	
2	1HPW/MMT	78	74	1.8	1.8
3	5HPW/MMT	76	76	1.9	0.38
4	10HPW/MMT	85	74	3.3	0.33
5	20HPW/MMT	91	68	5.5	0.28
6	1HPMo/MMT	81	72	1.7	1.7
7	5HPMo/MMT	88	69	2.4	0.48
8	10HPMo/MMT	93	64	4.4	0.44
9	20HPMo/MMT	97	54	6.2	0.31
10	HPW	27	0		0.9
11	HPMo	66	56		19.5
12	no catalyst	2	0		

This observation can be explained by partial pore blockage and reduced accessibility of active sites caused by heteropoly acid incorporation, as confirmed by the decrease in specific surface area and pore volume (BET data, Table 2). UV–vis diffuse reflectance spectra (Figure S7) provide further evidence: at high HPW loading, the characteristic montmorillonite band at ∼250 nm was largely masked by the HPW band at 265 nm, indicating extensive surface coverage.

We also tested the use of a homogeneous HPW (Table 4, row 10). Significantly lower conversion and only selectivity to undesired products were observed.

Using HPMo-modified montmorillonite (Figure S19 and Table 4, rows 6–9), a similar trend as in the case of HPW was observed: the increase of limonene conversion with increasing amount of HPMo content. Compared with HPW-modified materials, HPMo-modified materials provided slightly higher conversions. On the other hand, HPMo-modified materials provided lower selectivity, e.g., in the case of material modified with 20 wt % HPA—selectivity for HPW was 68% and HPMo was 54% (24 h). Moreover, the decrease of selectivity with increasing amount of HPMo was observed. This fact implicated that the catalytic activity of HPMo included also the increase of side reactions. An increase of initial reaction rate with HPMo content again has the same trend as in the case of HPW. Calculation of specific initial reaction rate values related to active substance (HPMo) led to values of 1.7 mmol/g·h for 1HPMo/MMT and 0.31 mmol/g·h for 20HPMo/MMT. The use of pure homogeneous HPMo (Table 4, row 11) resulted in a significantly higher reaction rate compared with HPMo supported on MMT. This behavior was anticipated due to the nature of the homogeneous catalyst. However, the heterogeneous HPMo/MMT catalyst exhibited higher selectivity toward the desired TPOBN product at all loadings, except the highest one.

Unlike acid-treated montmorillonites, which did not exhibit a clear relationship between acidity and catalytic performance, montmorillonites modified with heteropoly acids, phosphotungstic acid (HPW) and phosphomolybdic acid (HPMo), showed a distinct trend. An increase in total acidity was accompanied by a corresponding nonlinear enhancement in the initial reaction rate, as shown in Figure S20. This positive correlation indicates that the catalytic activity of HPW- and HPMo-modified montmorillonites is strongly governed by the additional acidity introduced through heteropoly acid incorporation. However, this increase in acidity was also associated with a slight decline in the product selectivity. This effect was particularly evident in the case of HPMo-treated montmorillonites, which exhibited the highest acidity among the tested samples. As shown in Figure S21, the selectivity decreased with increasing acidity, indicating that stronger acid sites may promote side reactions or alternative reaction pathways, thereby reducing the formation of the desired product. The decrease in selectivity was primarily associated with the formation of byproducts with a *p*-menthane structure derived from limonene. In the case of HPMo-supported catalysts, this effect was particularly pronounced, likely because the protonated limonene intermediate, common to both the desired product and these byproducts, becomes less stable and more prone to rearrangement under the strong acidity introduced by HPMo.

The comparative analysis of HPW and HPMo systems reveals that while both catalysts benefit from increased acidity in terms of activity, the impact on selectivity is more pronounced for HPMo. These findings underscore the dual role of acidity in catalytic systems (enhancing activity while potentially compromising selectivity) and highlight the need for careful optimization of acid strength to achieve balanced catalytic performance.

Heterogeneity of reaction with HPA-modified montmorillonites was confirmed because the possibility of HPA leaching to the reaction mixture was considered. No leaching of HPA to the reaction mixture was observed, meaning that the reaction was only heterogeneously catalyzed.

Comparing HPA-modified MMT and acid-treated MMT, we were able to decide that the use of only acid-treated MMT was more advisable. It was connected with a similar or better reaction course, and the selectivity and also the use of relatively expensive HPA were avoided.

### 2.4 Selectivity of Reaction and Reaction Mechanism

The main side products observed in reactions were mostly the products of limonene isomerization, *p*-methanic terpenes, terpinolene, α-terpinene, and γ-terpinene above all. Limonene isomerization can be explained by the fact that the carbenium ion intermediate can, in an acid environment, lose H^+^ before reacting with crotonaldehyde. Isomerization of limonene over montmorillonite is widely described in the literature.^47,48^ Products of crotonaldehyde aldol condensation or dimerization were not observed in the reaction mixtures.

2,2,6-Trimethyl-4-(1-propenyl)-3-oxabicyclo­[3.3.1]­non-6-ene (TPOBN) (iv) is synthesized by the Prins reaction of limonene (i) with crotonaldehyde (Figure 6). First step in the reaction mechanism, based on literature,^4,5,9^ is performed by protonation of the exocyclic limonene double bond, providing a carbocation intermediate (ii). This carbocation intermediate is subjected to nucleophilic attack of crotonaldehyde, providing the oxo-carbenium intermediate (iii). Following cyclization is performed by intramolecular nucleophilic attack of the limonene cyclic double bond to the oxo-carbenium ion. Carbocation can lose H^+^ before attack of crotonaldehyde, providing other *p*-menthanic terpenes, which were the proven side products in this reaction. Carbocation and oxo-carbenium ions can interact with other terpene molecules (limonene or others) to form products with higher molecular weight, therefore the molar excess of crotonaldehyde is preferred.^4,5,9^

**Figure 6:**
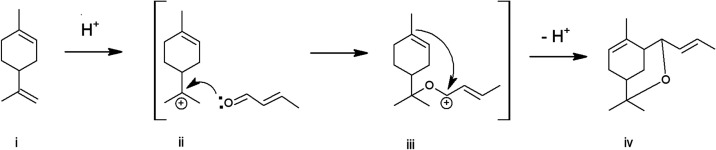
Simplified reaction mechanism.

### 2.5 Comparison with Data in Literature

Comparison with data for the Prins reaction of limonene with crotonaldehyde was performed (Table 5). Literature data for this reaction are only rare. Work of Costa^4^ presents the utilization of HPW-modified silica and cesium salt of phoshotungstic acid (CsPW). The only clay mentioned in the studied reaction was ascanite-bentonite clay,^9^ using which the results were significantly worse. From our work, we can conclude that acid-treated montmorillonite is able to provide quite high conversion and satisfactory selectivity and is worth noting that acid-treated materials are cheaper and more frequently used also on an industrial scale compared with HPA. A valuable finding is that toluene can also be used for this reaction, offering a more environmentally friendly alternative to commonly reported chlorinated solvents.

**Table 5:** Comparison with Data from the Literature

catalyst	reaction conditions	reaction result	ref
20%HPW/SiO_2_	50 °C, 5 h, 1 mmol of limonene, 3 mmol of crotonaldehyde, 30 mg of cat., 1,2-dichlorethane as solvent	94% limonene conversion, 86% selectivity	^4^
CsPW	70 °C, 5 h, 1 mmol of limonene, 3 mmol of crotonaldehyde, 30 mg of cat., 1,2-dichlorethane as solvent	95% limonene conversion, 90% selectivity	
HNO_3_/MMT	60 °C, 48 h, 1 mmol of limonene, 1.5 mmol of crotonaldehyde, 109 mg of cat., toluene as solvent	73% limonene conversion, 92% selectivity	this work
HNO_3_/MMT	60 °C, 24 h, 1 mmol of limonene, 1.5 mmol of crotonaldehyde, 109 mg of cat., toluene as solvent	95% limonene conversion, 70% selectivity	
10HPW/MMT		85% limonene conversion, 74% selectivity	
1HPMo/MMT		81% limonene conversion, 72% selectivity	
ascanite-bentonite clay	20 °C, 2 h, 0.54 mmol of limonene, 0.87 mmol of crotonaldehyde, 650 mg of cat., dichloromethane as solvent	yield 33%	^9^

## 3 Conclusion

Several types of acid-treated montmorillonites and montmorillonites modified with heteropoly acids were prepared and characterized. These materials have proven to be catalytically active in the Prins reaction of limonene with crotonaldehyde, giving 2,2,6-trimethyl-4-(1-propenyl)-3-oxabicyclo[3.3.1]­non-6-ene, a desired fragrance and pharmaceutically important bicyclic ether. All acid-treated materials provided higher conversion compared with that of commercially available montmorillonite. The increase of acidity of all prepared materials was connected with the formation of strong acid sites. The best obtained result was 73% limonene conversion and 92% selectivity to the desired product using HNO_3_/MMT, solvent toluene, and 60 °C after 48 h. The most important parameter of the catalyst for the highest activity was probably its specific surface. However, all acid-treated montmorillonites showed comparable activity. Reaction temperature showed to have a significant influence on the reaction course; higher reaction temperature provided higher conversion, but lower selectivity leading to an increase of the amount of *p*-menthanic products in the reaction mixture. The solvent choice for the reaction was shown to be crucial (as often in the reactions of terpenes); the best results were obtained using toluene; 1,4-dioxane provided also satisfactory results, but heptane and acetonitrile were not suitable solvents for the studied reaction. The main parameter of the solvent influencing the rate and selectivity seemed to be the solvent polarity. HPW- and HPMo-modified montmorillonites provided higher conversion compared with commercially available montmorillonite. The reaction result using HPW-modified materials was similar to acid-treated materials; on the other hand, HPMo materials provided higher limonene conversion, accompanied by lower selectivity. The decrease in selectivity at higher temperatures was primarily due to the isomerization of limonene, indicating a shift from kinetic to thermodynamic control under conditions that favor stronger acid sites and less stable protonated intermediates. Appropriate modification of montmorillonite showed a positive effect on the synthesis of the desired bicyclic ether. Our findings clearly demonstrate that the appropriate simple modification of montmorillonite significantly enhances its catalytic performance and selectivity, offering promising potential for the efficient synthesis of pharmaceutically valuable bicyclic ethers.

## 4 Experimental Section

### 4.1 Material Synthesis

#### 4.1.1 Acid-Treated Materials

Montmorillonite K10 (1 g, Sigma-Aldrich) was mixed with 5 mL of 1 M acid water solution. The mass ratio of 1 M acid to montmorillonite was 5:1. The suspension was stirred at room temperature (RT) overnight, filtered (filter S4), and washed with demineralized water until the filtrate was neutral. Prepared materials were dried in an oven under air at 100 °C. Acids used for this modification were nitric acid (65%, Penta), sulfuric acid (96%, Penta), hydrochloric acid (35%, Penta), and phosphoric acid (85%, Penta). Materials were denoted with the type of acid used for treatment, e.g., HCl/MMT means material treated with hydrochloric acid.

#### 4.1.2 Heteropoly Acid (HPA)-Modified Materials

HPA-modified materials were prepared by the wet impregnation method. Montmorillonite K10 (1 g, Sigma-Aldrich) was mixed with 5 mL of HPA water solution containing the desired calculated amount of HPA. The suspension was stirred at room temperature (RT) for 3 h, and then water was evaporated using a rotary evaporator. Prepared materials were dried in an oven under air at 100 °C and homogenized in a mortar. HPA used for impregnation was phosphomolybdic acid hydrate (HPMo) and phosphotungstic acid hydrate (HPW, both from Sigma-Aldrich). Materials were denoted according to wt % HPA used, e.g., 1HPW/MMT means 1 wt % HPW on MMT.

### 4.2 Catalytic Testing

#### 4.2.1 Typical Experiment

Prins reaction of limonene with crotonaldehyde was performed in a round-bottomed flask with a Liebig condenser—the flask was filled with solvent (3 mL), D-/L-limonene (1:1 mixture) (1 mmol, Merck, >95%), crotonaldehyde (1.5 mmol, Sigma-Aldrich, 98%), and the catalyst. The reaction mixture was stirred continuously (700 rpm) at different reaction temperatures (40–80 °C) for 24 h. Samples taken from the reaction mixture were analyzed using a gas chromatograph equipped with a VF-5 column and a flame ionization detector. Solvents used for the reaction were 1,4-dioxane, toluene, heptane, and acetonitrile (all Penta, p.a.). An internal standard (*p*-xylene, Sigma-Aldrich, 99%) was used. The structures of side products were confirmed by comparing the retention times with commercial standards and comparing the spectra obtained by GC-MS analysis (nonpolar column). The GC-MS spectrum of the product was compared with the MS spectrum presented in the literature.^4^

When performing the reaction with nonsupported (pure) HPAs, HPA amounts corresponding to the amount of HPA in 10HPMo/MMT or 10HPW/MMT were used.

#### 4.2.2 Heterogeneity of Reaction

Heterogeneity of the reaction arrangement was confirmed by the hot filtration test: the reaction was performed as usual, and after 1 h of reaction, the catalyst was filtered from the reaction mixture (syringe filter 0.45 μm), and the reaction continued at a specified temperature. No reaction occurred in any case after catalyst removal, meaning that the reaction was heterogeneously catalyzed.

#### 4.2.3 Reuse Experiment

The reaction mixture was centrifuged, and the catalyst was separated and washed three times with toluene, one time with ethanol (Penta, 96%), and dried in an oven under air at 100 °C; this process provided a recycled catalyst. To obtain a regenerated catalyst, the recycled catalyst was calcined at 500 °C (air, 12 h) and subsequently acid treated as described in Section Acid-Treated Materials. Calcination in the regeneration process was used to remove all carbonaceous deposits, which occurred on the material surface during the first cycle of the reaction. Following acid treatment led to the restoration of the montmorillonite structure destroyed during calcination. A recycled or regenerated catalyst was used in the reaction as usual according to Section Typical Experiment.

### 4.3 Characterization Techniques

X-ray fluorescence (XRF) analysis was performed on a WD-XRF ARL PERFORM’X Spectrometer (Thermo Scientific).

Diffuse reflectance UV–vis measurement was performed using a Shimadzu 2600i spectrophotometer equipped with an integrating sphere ISR-2600 Plus. The solid samples were measured on BaSO_4_ tablets in the wavelength range of 220–1400 nm.

Structural characterization of catalysts by ATR-IR was performed on a Nicolet iS50 instrument with a resolution of 4 cm^–1^ and a scan count of 64 scans in the wavenumber range of 4000–500 cm^–1^.

Nitrogen adsorption was measured using a 3Flex volumetric analyzer (Micromeritics). Specific surface area was calculated via the BET equation, and total pore volume was calculated using the *t*-plot method.

Temperature-programmed oxidation (TPO) was performed to analyze the amount of carbonaceous deposits on the reused catalysts (Autochem III, Micromeritics). Both a thermal conductivity detector (TCD) and a quadrupole mass spectrometer (MKS Cirrus 2 Analyzer) with a capillary coupling system were used for the CO_2_ detection (released after the oxidation of carbonaceous deposits from the samples). A material sample (0.05 g) was placed in a quartz U-shaped tube. Before the TPO experiment, the catalyst was heated under a helium flow (30 mL/min) up to 80 °C, and kept at 80 °C for 30 min to dry the catalyst. Afterward, a catalyst was set up to O_2_–He flow (2% O_2_ in helium, 30 mL/min), the linear temperature program (15 °C/min) started, and the sample was heated up to a temperature of 900 °C. The amount of originated carbon dioxide was determined by calibrating the intensity of the 44 amu MS response (0.5 mL loop).^13,18^

Temperature-programmed desorption (TPD) of ammonia was performed to compare the acidity of the materials (AutoChem III, Micromeritics). A thermal conductivity detector (TCD) was used for desorbed ammonia detection. A catalyst sample (0.1 g) was placed in a U-shaped quartz tube. Prior to the adsorption of ammonia, the catalyst was heated under a helium flow (25 mL/min) up to 100 °C and kept at 100 °C for 60 min to remove impurities from the sample and activate the material surface. The sample was cooled to 50 °C and exposed to ammonia flow (25 mL/min) for 30 min to saturate the material. Then, the sample was flushed with helium for 60 min to remove the physisorbed ammonia. Afterward, the linear temperature program (10 °C/min) was started at a temperature of 50 °C, and the sample was heated up to a temperature of 800 °C. The amount of desorbed ammonia was determined by calibration using a TCD detector (0.5 mL loop). A cool trap (cooled by acetone with dry ice) was used to remove water from the helium stream.^49,50^ Temperature-programmed desorption spectra revealed two peaks: first, a broad peak with maxima around 120 °C was considered as weak and moderate acid sites, and second, with maxima around 550 °C was considered as strong acid sites.

### 4.4 Calculations

Initial reaction rate (*u*_1h_) was calculated via eq 1, where *m*_cat_ is the amount of catalyst entering the reaction, *n*_lim,0_ is the molar amount of limonene entering reaction in time *t* = 0, *t* is the time of sample withdrawal (1 h), and ζ_1h_ is the conversion of limonene in time of sample withdrawal (*t* = 1 h).

1 $$u_{1h}=\frac{\zeta_{1h}\cdot n_{\lim,0}}{m_{\mathrm{cat}}\cdot t}$$

Specific initial reaction rate (*r*_1h_) was calculated in the case of HPA-modified materials via eq 2, where *u*_1h_ is the initial reaction rate calculated via eq 1, and *w*_HPA_ is the wt % of HPA on the catalyst (e.g., for 10HPW/MMT *w*_HPW_ = 10).

2 $$r_{1h}=\frac{u_{1h}}{w_{\mathrm{HPA}}}$$

## Supplementary Material



## Notes

Supporting figures for nitrogen physisorption of prepared materials, dependence of specific surface area on the Si/Al ratio, temperature-programmed desorption spectra and UV–vis spectra of HPA-modified materials, ATR-FTIR spectra for prepared materials, figures for reaction course at different reaction conditions, and dependence of reaction rate or selectivity on material acidity (PDF)
