# Supplementary material for: Montmorillonite—Eco-friendly and Effective Catalyst in the Synthesis of Biologically Active Compounds with Bicyclo[3.3.1] Moiety
Source: ACS Omega. 2025 Dec 13;10(51):63597–607. doi: 10.1021/acsomega.5c10876 (PMC12756779; doi:10.1021/acsomega.5c10876)

Montmorillonite – eco-friendly and effective catalyst in synthesis of biologically active compounds with bicyclo[3.3.1] moiety

Eva Vrbková*, Lucie Stoupová, Eliška Vyskočilová

Dpt. of organic technology, University of Chemistry and Technology Prague, Technická 6, Prague, Czech Republic
*eva.vrbkova@vscht.cz

Figure S1. Nitrogen physisorption results for acid treated montmorillonites

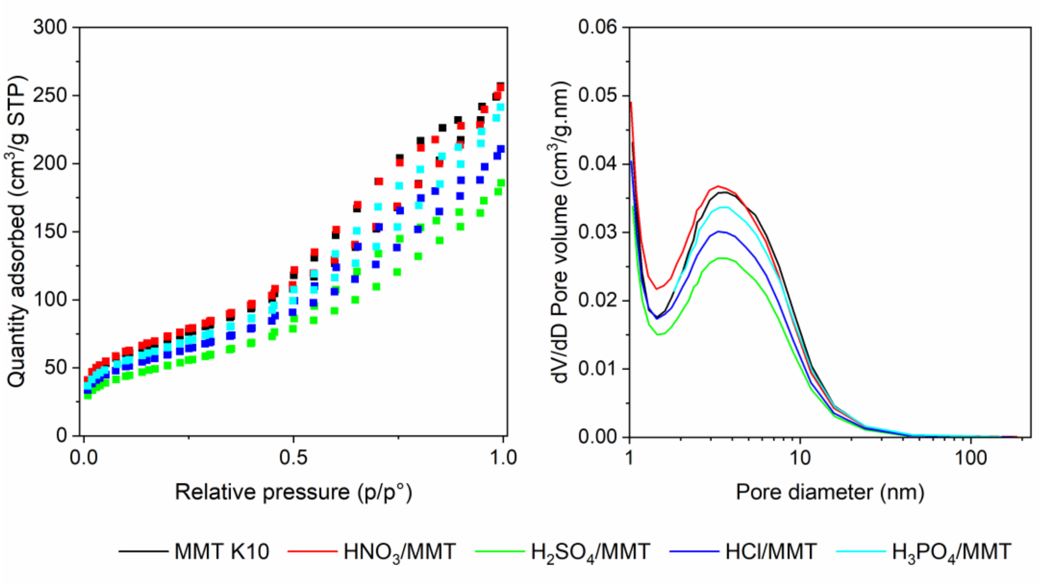

Figure S2. Nitrogen physisorption results for HPW modified montmorillonites

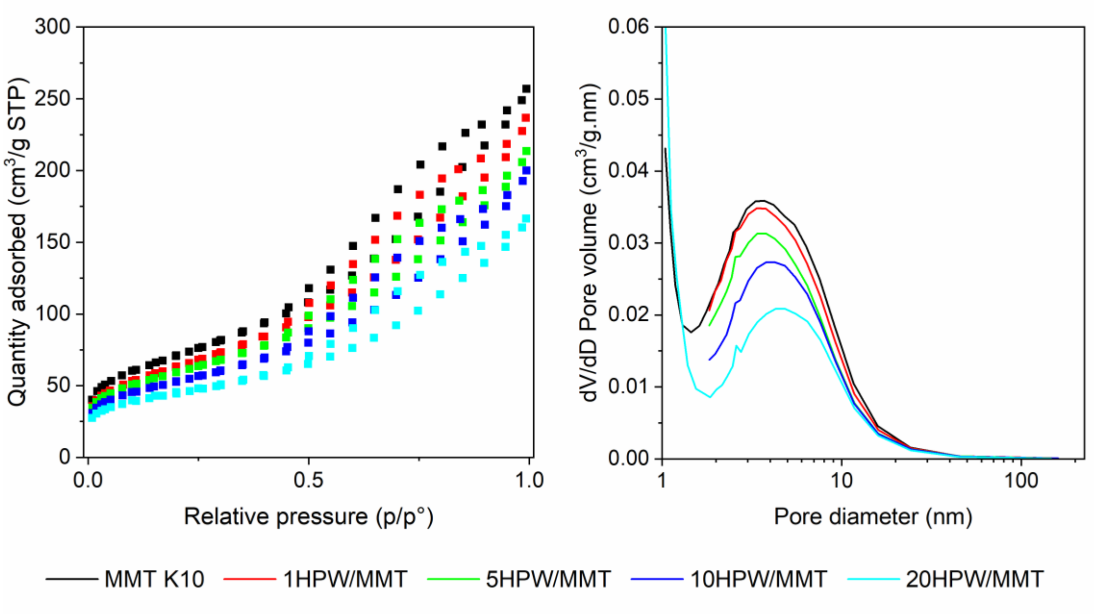

Figure S3. Nitrogen physisorption results for HPMo modified montmorillonites

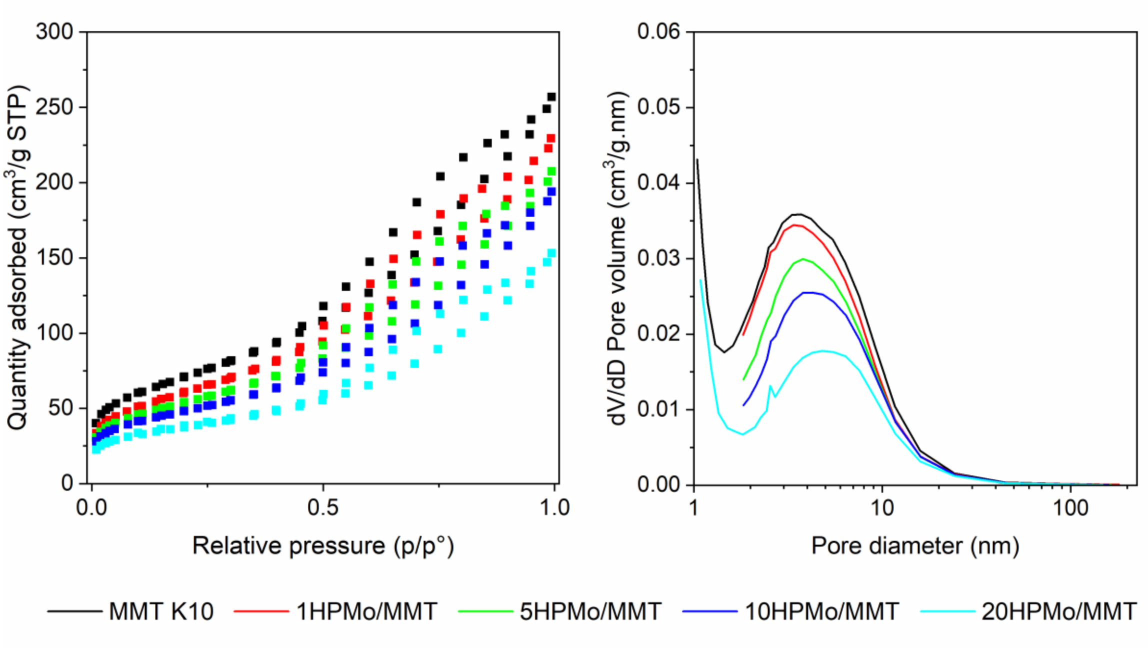

Figure S4. Specific surface area dependent on Si/Al ratio

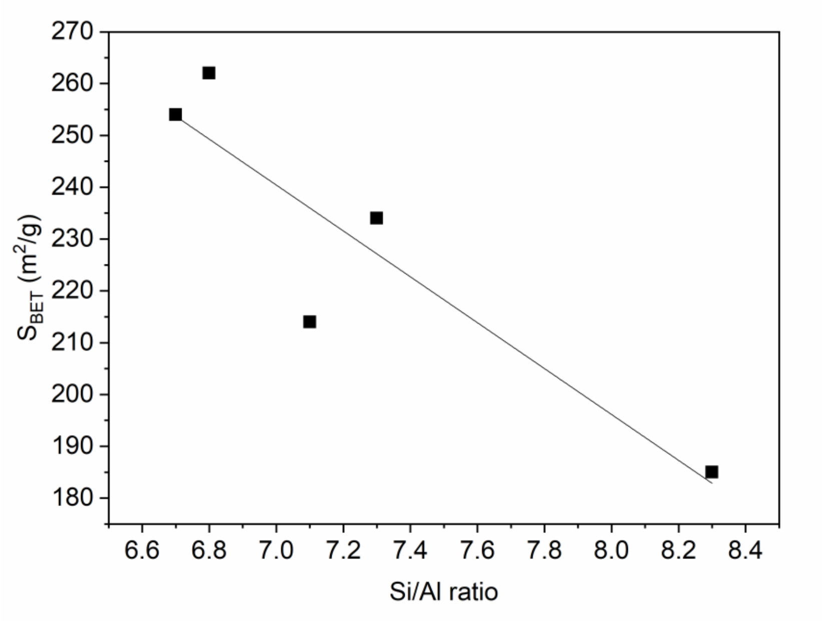

Figure S5. Temperature programmed desorption of ammonia for HPW modified montmorillonites

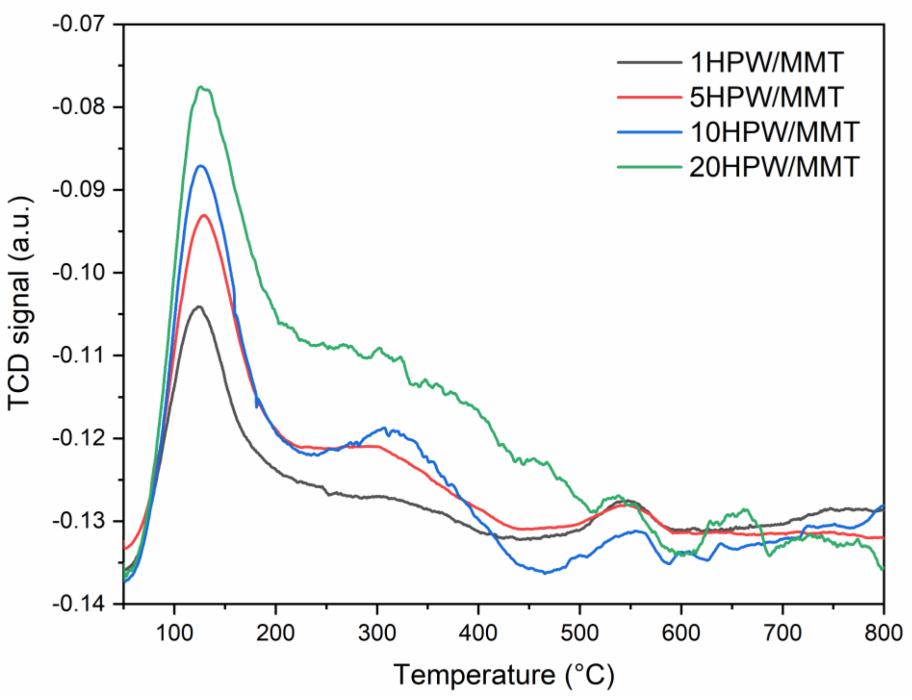

Figure S6. Temperature programmed desorption of ammonia for HPMo modified montmorillonites

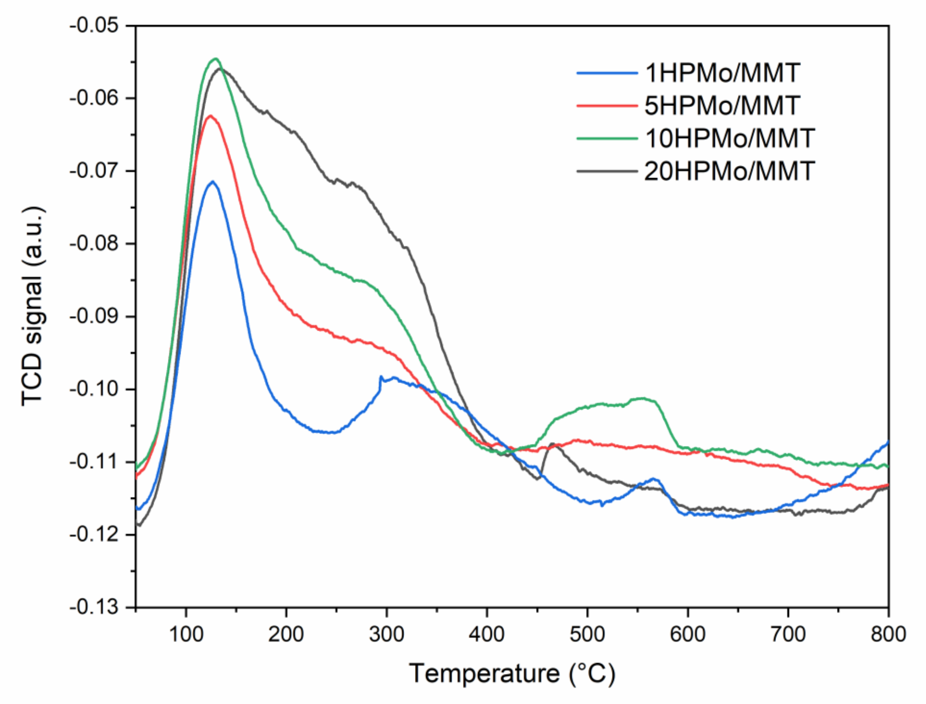

Figure S7. UV-Vis spectrum of materials modified with HPW (left) or HPMo (right) (● MMT, ● 1% HPA/MMT, ● 5% HPA/MMT, ● 10% HPA/MMT, ● 20% HPA/MMT)

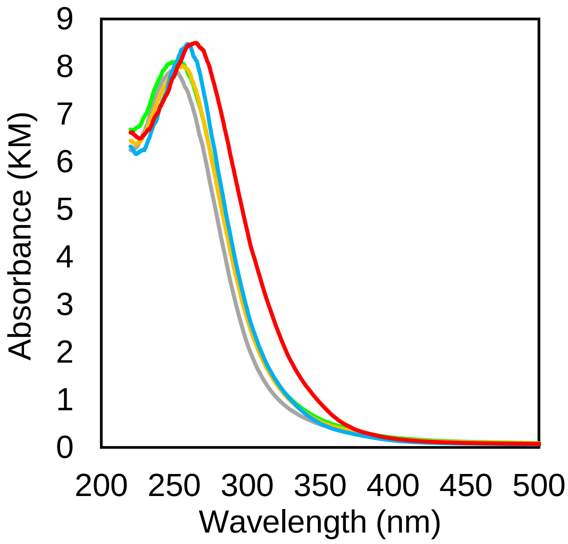

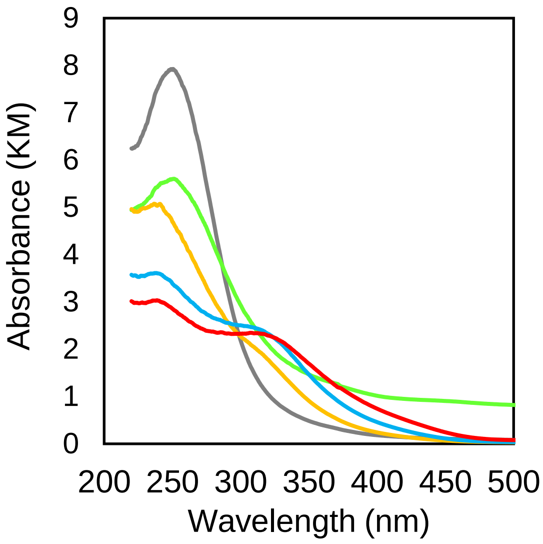

Figure S8. ATR-FTIR spectrum of acid treated montmorillonites

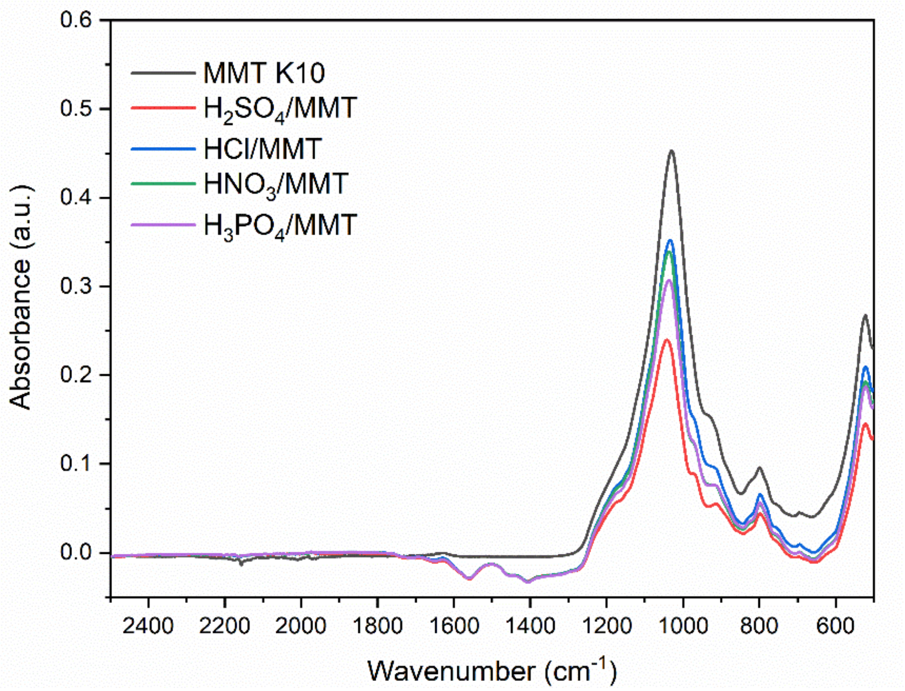

Figure S9. ATR-FTIR spectrum of montmorillonites modified with HPMo

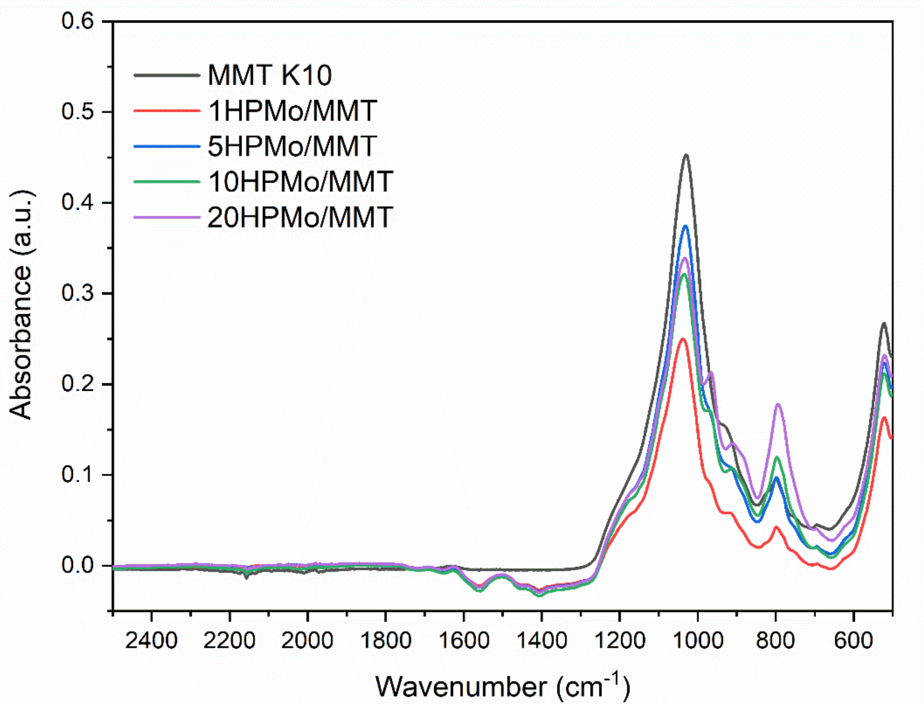

Figure S10. ATR-FTIR spectrum of montmorillonites modified with HPW

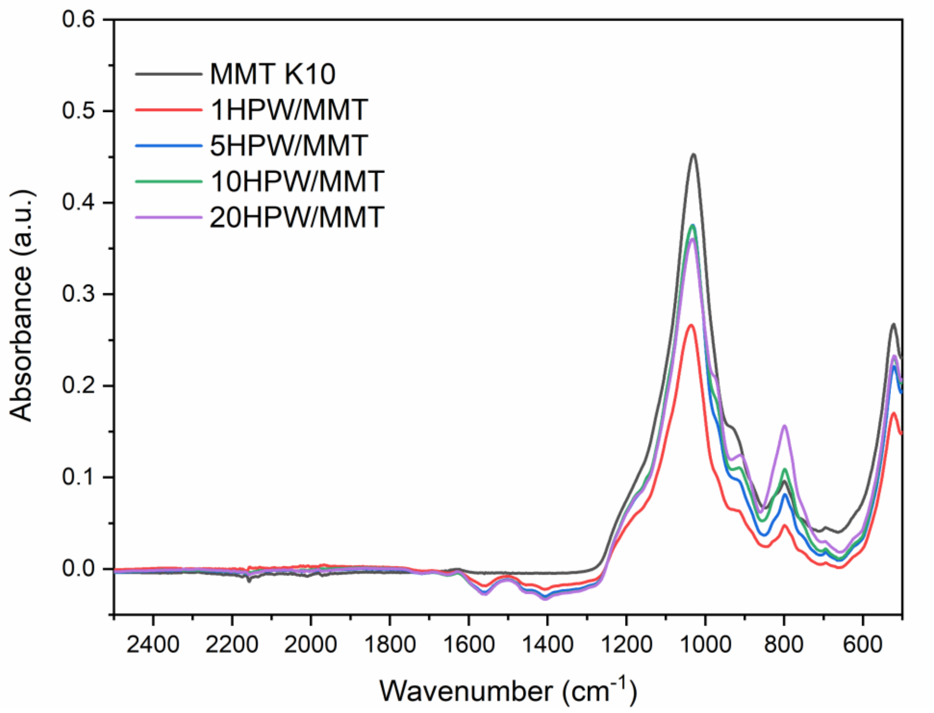

Figure S11. Reaction course in case of reaction time 48 h (60 °C, 80 wt.% of cat. HNO_3/MMT to limonene, molar ratio L:C = 1:1.5, toluene)

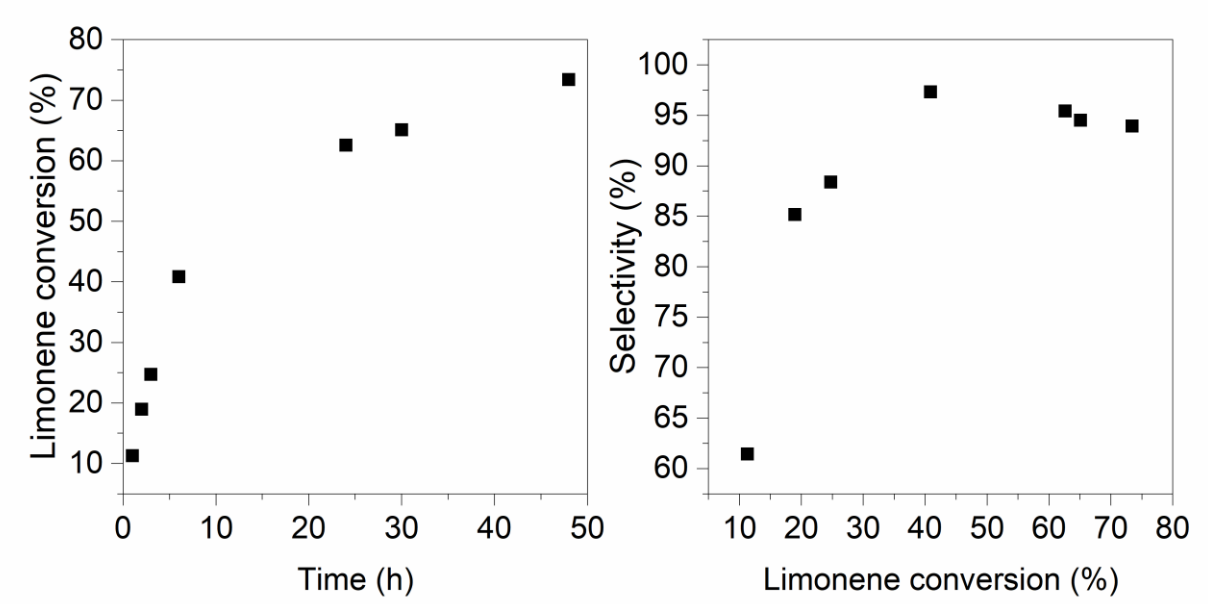

Figure S12. Reaction course in case of using different solvents (60 °C, 40 wt.% of cat. H_2SO_4/MMT to limonene, molar ratio L:C = 1:1.5)

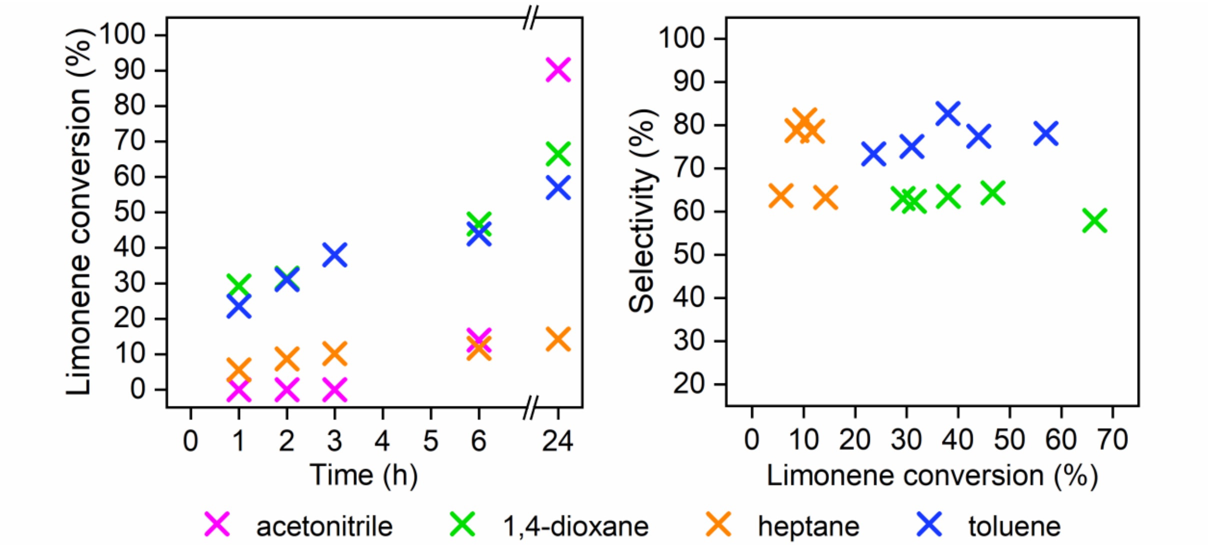

Figure S13. Reaction course in case of using catalyst amount (toluene, 60 °C, cat. H_2SO_4/MMT, molar ratio L:C = 1:1.5)

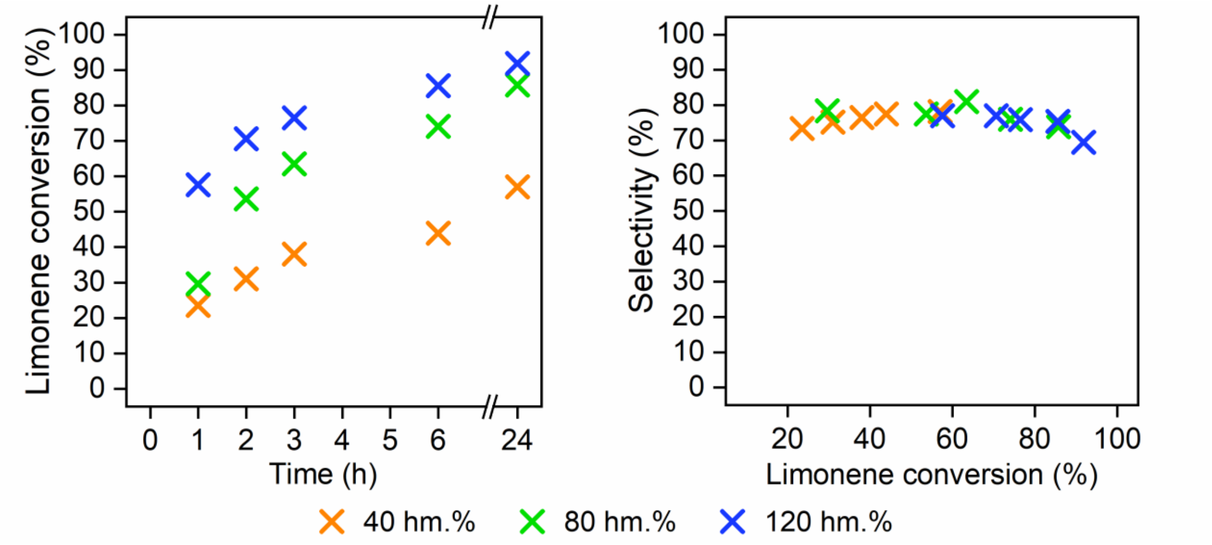

Figure S14. Reaction course in case of using different molar ratio L:C (toluene, 60 °C, 40 wt.% of cat. H_2SO_4/MMT to limonene)

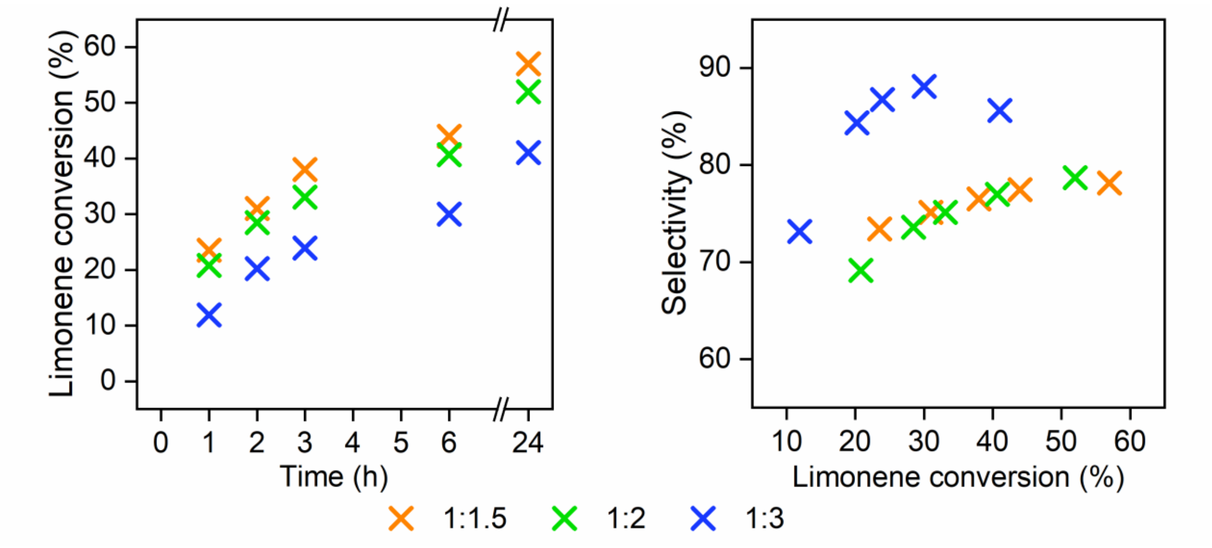

Figure S15. Initial reaction rate dependent on total material acidity (left) and on weak and medium acid sites (right) (acid-treated montmorillonites)

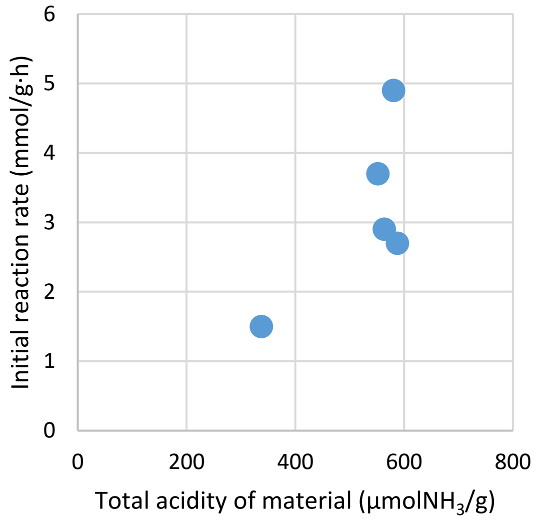

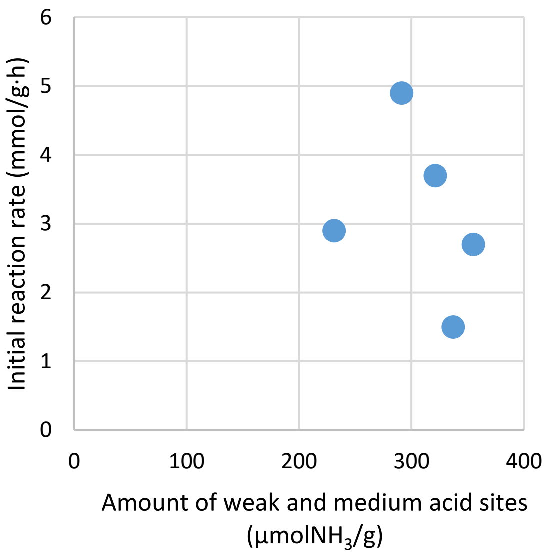

Figure S16. Selectivity dependent on weak and medium acid sites (acid-treated montmorillonites)

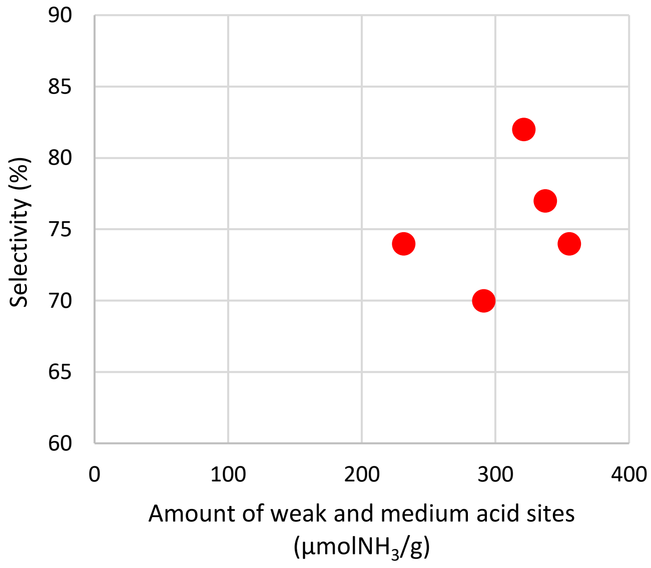

Figure S17. Reaction course in case of using recycled catalyst (toluene, 60 °C, 80 wt.% of cat. HNO_3/MMT to limonene, molar ratio L:C = 1:1.5)

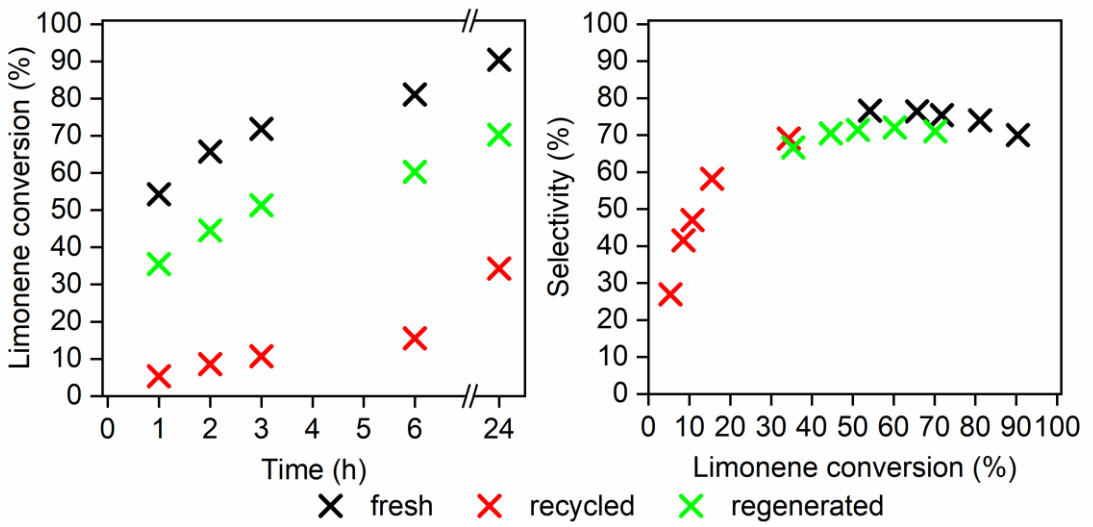

Figure S18. Reaction course in case of using HPW modified montmorillonites (toluene, 60 °C, 80 wt.% of cat. to limonene, molar ratio L:C = 1:1.5)

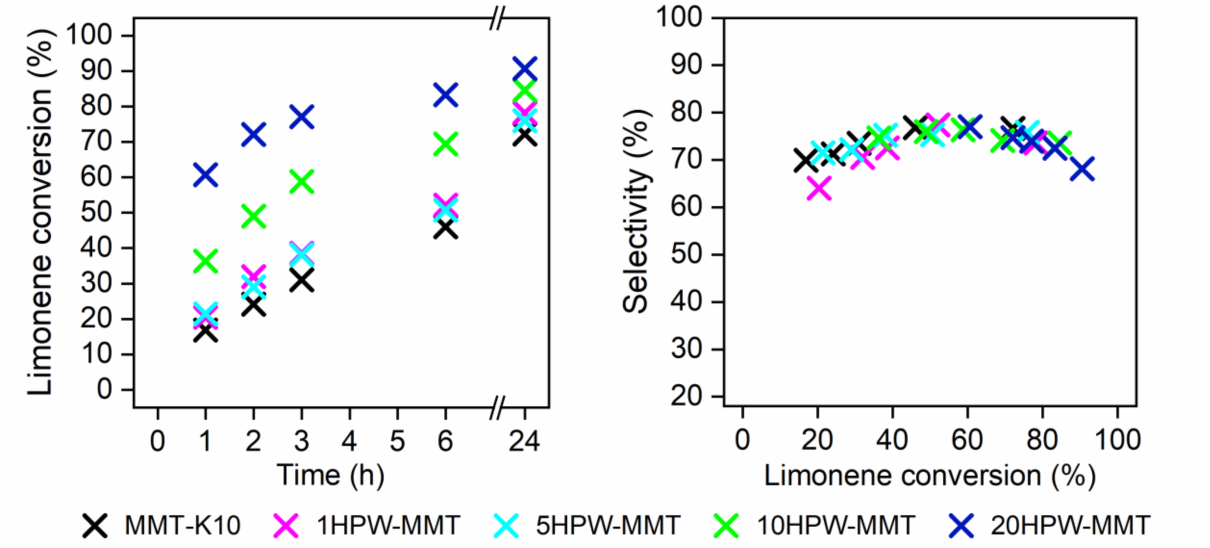

Figure S19. Reaction course in case of using HPMo modified montmorillonites (toluene, 60 °C, 80 wt.% of cat. to limonene, molar ratio L:C = 1:1.5)

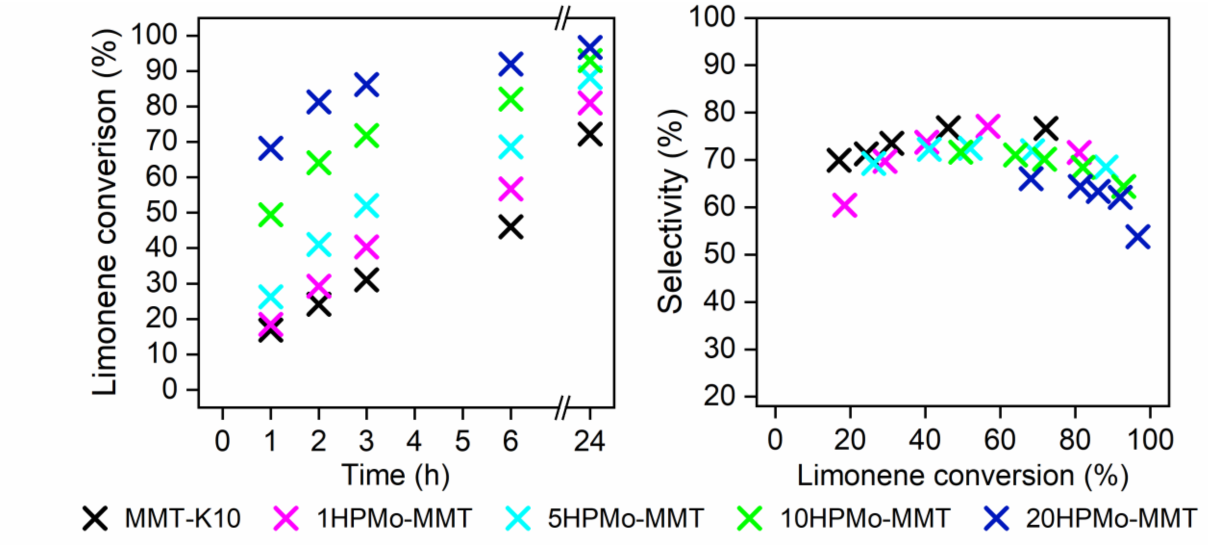

Figure S20. Initial reaction rate dependent on total material acidity (left – HPMo modified materials, right – HPW modified materials)

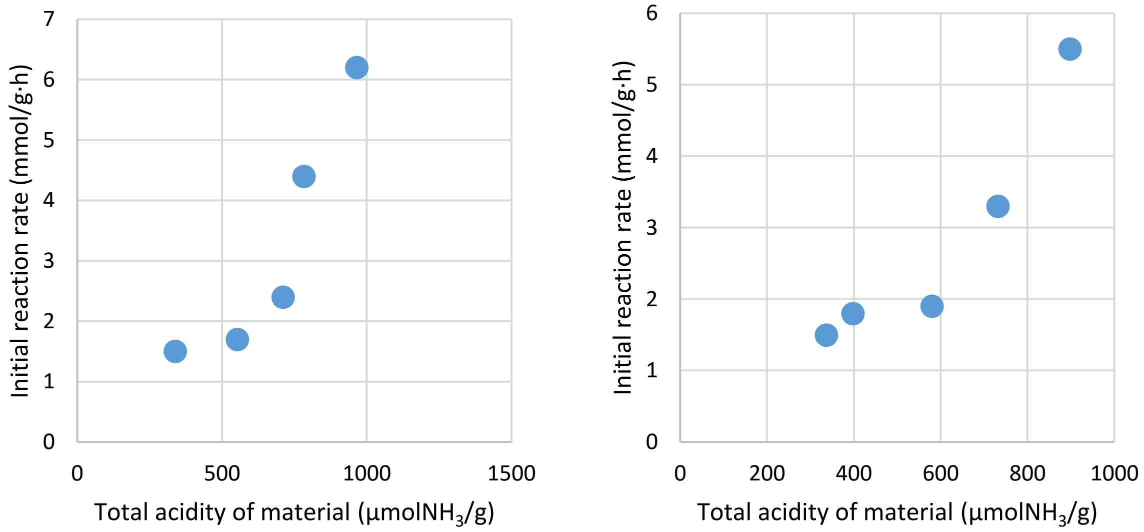

Figure S21. Selectivity dependent on total material acidity (left – HPMo modified materials, right – HPW modified materials)

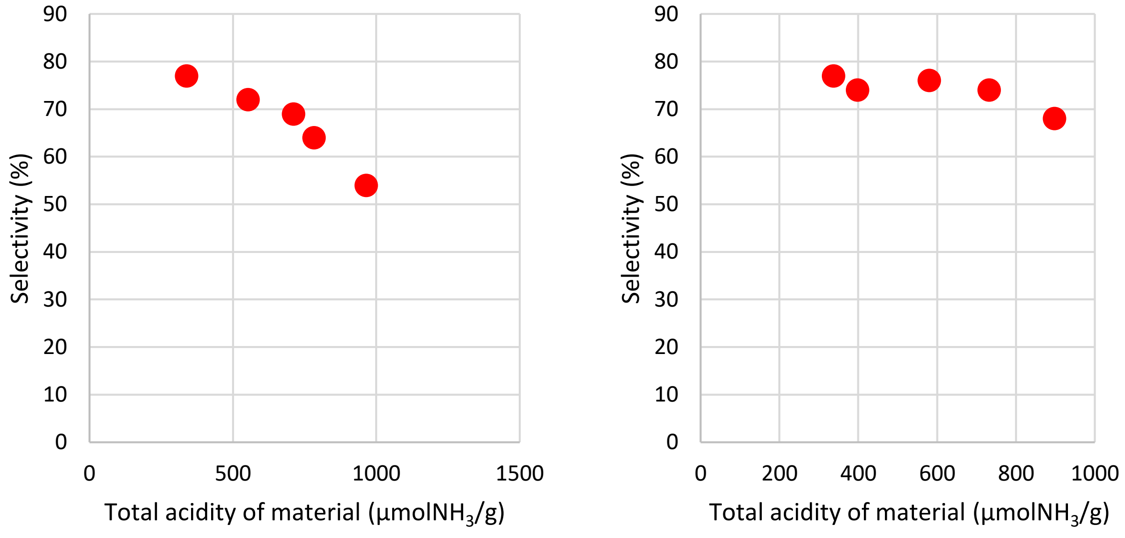

Supplement: Supplementary file 1 [file ao5c10876_si_001.pdf]
